# Supplementary material for: Mental healthcare expenditure among adults by type of mental healthcare and its association with age and sex in the Netherlands between 2015 and 2020
Source: PLoS One. 2025 Aug 25;20(8):e0330365. doi: 10.1371/journal.pone.0330365 (PMC12377576; doi:10.1371/journal.pone.0330365)
Supplement: S1 File — (PDF) [file pone.0330365.s001.pdf]

Table of contents

S1 Regression output for total mental healthcare costs and age 2

S2 Predicted costs by agegroup for total mental healthcare costs 4

S3 Marginal estimates for the association between total mental healthcare costs and young age 5

S4 Regression output for SPECIALIST-MHC and age 6

S5 Predicted costs by agegroup for SPECIALIST-MHC costs 8

S6 Marginal estimates for the association between SPECIALIST-MHC costs and young age 9

S7 Regression output for BASIC-MHC costs and age 10

S8 Predicted costs by agegroup for BASIC-MHC costs 12

S9 Marginal estimates for the association between BASIC-MHC costs and young age 13

S10 Regression output for GP-MHC costs and age 14

S11 Predicted costs by agegroup for GP-MHC costs 16

S12 Marginal estimates for the association between GP-MHC costs and young age 17

S13 Regression output for total mental healthcare costs and sex 18

S14 Predicted costs by sex for total mental healthcare costs 20

S15 Regression output for SPECIALIST-MHC costs and sex 21

S16 Predicted costs by sex for SPECIALIST-MHC costs 23

S17 Regression output for BASIC-MHC costs and sex 24

S18 Predicted costs by sex for BASIC-MHC costs 26

S19 Regression output for GP-MHC costs and sex 27

S20 Predicted costs by sex for GP-MHC costs 29

S21 Regression output for total mental healthcare costs, age and sex 30

S22 Marginal estimates for the association between age and total mental healthcare costs by sex 32

S23 Regression output for SPECIALIST-MHC costs, age and sex 33

S24 Marginal estimates for the association between age and SPECIALIST-MHC costs by sex 35

S25 Regression output for BASIC-MHC costs, age and sex 36

S26 Marginal estimates for the association between age and BASIC-MHC costs by sex 38

S27 Regression output for GP-MHC costs, age and sex 39

S28 Marginal estimates for the association between age and GP-MHC costs by sex 41

S1 Regression output for total mental healthcare costs and age

	Coefficient	Robust std. err.	t	P>|t|	[95% conf. interval]	
Sex	0.37887	0.011128	34.05	0	0.357054	0.400687
Age						
18-34 years	0.284951	0.019718	14.45	0	0.246292	0.32361
Year						
2016	0.032476	0.002742	11.84	0	0.0271	0.037852
2017	0.07188	0.004146	17.34	0	0.063752	0.080007
2018	0.129545	0.006012	21.55	0	0.117759	0.141331
2019	0.171828	0.007842	21.91	0	0.156454	0.187203
2020	0.204271	0.007623	26.8	0	0.189326	0.219216
age#year						
18-34 years#2016	0.006417	0.005294	1.21	0.226	-0.00396	0.016796
18-34 years#2017	0.034743	0.007239	4.8	0	0.02055	0.048936
18-34 years#2018	0.079937	0.008758	9.13	0	0.062767	0.097107
18-34 years#2019	0.139272	0.009066	15.36	0	0.121498	0.157046
18-34 years#2020	0.194877	0.007062	27.6	0	0.181032	0.208722
constant	-205.919	0.030697	-6708.13	0	-205.98	-205.859

Regression output for the linear regression of mental healthcare costs and age weighted for the number of insured years per unit of analysis and accounting for sex, year, the interactions between age and year, and for clustering by 4-number postal code.

	Coefficient	Robust std. err.	t	P>|t|	[95% conf. interval]	
Urbanicity						
2	0.124135	0.015685	7.91	0	0.093383	0.154887
3	0.254126	0.018249	13.93	0	0.218347	0.289905
4	0.397518	0.016249	24.46	0	0.365659	0.429376
5	0.521654	0.020756	25.13	0	0.48096	0.562349
SES						
2	-0.18913	0.016603	-11.39	0	-0.22168	-0.15658
3	-0.35601	0.017249	-20.64	0	-0.38983	-0.32219
4	-0.44774	0.017157	-26.1	0	-0.48138	-0.4141
5	-0.55179	0.018149	-30.4	0	-0.58738	-0.51621
Sex	0.365926	0.003496	104.67	0	0.359072	0.372781
Age						
18-34 years	0.228283	0.007856	29.06	0	0.21288	0.243686
Year						
2016	0.027266	0.002751	9.91	0	0.021872	0.03266
2017	0.062965	0.003383	18.61	0	0.056331	0.069598
2018	0.126516	0.003856	32.81	0	0.118956	0.134075
2019	0.184374	0.004232	43.57	0	0.176076	0.192671
2020	0.218286	0.004666	46.79	0	0.209139	0.227433
age#year						
18-34 years#2016	0.01047	0.004992	2.1	0.036	0.000682	0.020258
18-34 years#2017	0.045508	0.005733	7.94	0	0.034269	0.056747
18-34 years#2018	0.094215	0.006064	15.54	0	0.082326	0.106104
18-34 years#2019	0.158598	0.006545	24.23	0	0.145766	0.17143
18-34 years#2020	0.208355	0.006853	30.4	0	0.194919	0.221791
constant	-205.935	0.019535	-11000	0	-205.973	-205.896

Regression output for the linear regression of mental healthcare costs and age weighted for the number of insured years per unit of analysis and accounting for urbanicity, sex, year, SES, the interactions between age and year, and for clustering by 4-number postal code.

S2 Predicted costs by agegroup for total mental healthcare costs

	Age 18-35	Age 35-65
	Marginal estimate (95%-CI)	
2015	-205.31 (-205.32;-205.30)	-205.08 (-205.10;-205.07)
2016	-205.05 (-205.06;-205.03)	-205.28 (-205.30;-205.27)
2017	-204.98 (-204.99;-204.96)	-205.25 (-205.26;-205.24)
2018	-204.86 (-204.88;-204.85)	-205.19 (-205.20;-205.17)
2019	-204.74 (-204.76;-204.72)	-205.13 (-205.14;-205.11)
2020	-204.66 (-204.67;-204.64)	-205.09 (-205.11;-205.08)

Marginal estimates for mental healthcare costs by agegroup per year. As plotted in figure 1.

S3 Marginal estimates for the association between total mental healthcare costs and young age

	Marginal estimate (95%-CI)
2015	0.23 (0.21; 0.24)
2016	0.24 (0.22; 0.25)
2017	0.27 (0.26; 0.29)
2018	0.32 (0.31; 0.34)
2019	0.39 (0.37; 0.40)
2020	0.44 (0.42; 0.45)

Marginal estimates for the association between mental healthcare costs and age per year. As plotted in figure 2.

S4 Regression output for SPECIALIST-MHC and age

	Coefficient	Robust std. err.	t	P>|t|	[95% conf. interval]	
Sex	0.333455	0.010493	31.78	0	0.312883	0.354027
Age						
18-34 years	0.283896	0.018685	15.19	0	0.247262	0.32053
Year						
2016	0.025182	0.002894	8.7	0	0.019508	0.030857
2017	0.061014	0.004172	14.63	0	0.052836	0.069193
2018	0.119344	0.005885	20.28	0	0.107806	0.130881
2019	0.16096	0.007509	21.43	0	0.146237	0.175683
2020	0.200443	0.007553	26.54	0	0.185635	0.215252
age#year						
18-34 years#2016	0.008436	0.005626	1.5	0.134	-0.00259	0.019466
18-34 years#2017	0.03776	0.007502	5.03	0	0.023053	0.052467
18-34 years#2018	0.083712	0.008983	9.32	0	0.066101	0.101323
18-34 years#2019	0.144964	0.009564	15.16	0	0.126214	0.163714
18-34 years#2020	0.203466	0.00775	26.25	0	0.188272	0.218659
constant	-198.781	0.028915	-6874.65	0	-198.838	-198.725

Regression output for the linear regression of mental healthcare costs and age weighted for the number of insured years per unit of analysis and accounting for sex, year, the interactions between age and year, and for clustering by 4-number postal code.

	Coefficient	Robus std. err.	t	P>|t|	[95% conf. interval]	
Urbanicity						
2	0.128316	0.016379	7.83	0	0.096202	0.160429
3	0.256157	0.019004	13.48	0	0.218898	0.293416
4	0.403467	0.017013	23.71	0	0.370111	0.436824
5	0.53913	0.021858	24.66	0	0.496274	0.581986
SES						
2	-0.20571	0.017608	-11.68	0	-0.24023	-0.17119
3	-0.38087	0.018168	-20.96	0	-0.41649	-0.34525
4	-0.47335	0.01813	-26.11	0	-0.5089	-0.43781
5	-0.588	0.01868	-31.48	0	-0.62462	-0.55137
Sex	0.321346	0.003795	84.69	0	0.313907	0.328786
Age						
18-34 years	0.221819	0.008366	26.52	0	0.205417	0.238221
Year						
2016	0.019577	0.002941	6.66	0	0.013811	0.025344
2017	0.051146	0.003656	13.99	0	0.043977	0.058315
2018	0.115363	0.004218	27.35	0	0.107094	0.123632
2019	0.172967	0.004625	37.4	0	0.163899	0.182035
2020	0.214438	0.005099	42.05	0	0.20444	0.224436
age#year						
18-34 years#2016	0.012386	0.005409	2.29	0.022	0.00178	0.022991
18-34 years#2017	0.048611	0.006221	7.81	0	0.036414	0.060809
18-34 years#2018	0.098277	0.006584	14.93	0	0.085368	0.111186
18-34 years#2019	0.165146	0.007125	23.18	0	0.151176	0.179116
18-34 years#2020	0.218278	0.007411	29.46	0	0.203749	0.232807
Constant	-198.788	0.020682	-9611.68	0	-198.829	-198.747

Regression output for the linear regression of mental healthcare costs and age weighted for the number of insured years per unit of analysis and accounting for urbanicity, sex, year, SES, the interactions between age and year, and for clustering by 4-number postal code.

S5 Predicted costs by agegroup for SPECIALIST-MHC costs

	Age 18-35	Age 35-65
	Marginal estimate (95%-CI)	
2015	-198.02 (-198.03;-198.00)	-198.24 (-198.25;-198.23)
2016	-197.99 (-198.00;-197.97)	-198.22 (-198.23;-198.21)
2017	-197.92 (-197.94;-197.90)	-198.20 (-198.20;-198.18)
2018	-197.81 (-197.82;-197.79)	-198.13 (-198.14;-198.11)
2019	-197.68 (-197.70;-197.66)	-198.07 (-198.08;-198.05)
2020	-197.59 (-197.60;-197.57)	-198.03 (-198.04;-198.01)

Marginal estimates for mental healthcare costs by agegroup per year. As plotted in figure 1.

S6 Marginal estimates for the association between SPECIALIST-MHC costs and young age

	Marginal estimate (95%-CI)
2015	0.22 (0.20; 0.24)
2016	0.23 (0.22; 0.25)
2017	0.27 (0.25; 0.28)
2018	0.32 (0.30; 0.33)
2019	0.38 (0.36; 0.40)
2020	0.44 (0.42; 0.45)

Marginal estimates for the association between mental healthcare costs and age per year. As plotted in figure 2.

S7 Regression output for BASIC-MHC costs and age

	Coefficient	Robust std. err.	t	P>|t|	[95% conf. interval]	
Sex	0.191571	0.002887	66.36	0	0.185911	0.197231
Age						
18-34 years	0.110504	0.00715	15.45	0	0.096485	0.124523
Year						
2016	0.027728	0.003266	8.49	0	0.021326	0.034131
2017	0.035992	0.003667	9.82	0	0.028803	0.043181
2018	0.04129	0.003939	10.48	0	0.033567	0.049012
2019	0.048307	0.004244	11.38	0	0.039987	0.056627
2020	0.025308	0.004283	5.91	0	0.016911	0.033705
age#year						
18-34 years#2016	-0.00731	0.004282	-1.71	0.088	-0.01571	0.001083
18-34 years#2017	-0.0076	0.004505	-1.69	0.092	-0.01643	0.001234
18-34 years#2018	0.004182	0.004936	0.85	0.397	-0.0055	0.01386
18-34 years#2019	0.020281	0.004386	4.62	0	0.011682	0.02888
18-34 years#2020	0.037068	0.004552	8.14	0	0.028143	0.045993
constant	-2.31817	0.008686	-266.88	0	-2.3352	-2.30114

Regression output for the linear regression of mental healthcare costs and age weighted for the number of insured years per unit of analysis and accounting for sex, year, the interactions between age and year, and for clustering by 4-number postal code.

	Coefficient	Robust std. err.	t	P>|t|	[95% conf. interval]	
Urbanicity						
2	0.195795	0.022901	8.55	0	0.150894	0.240697
3	0.341027	0.023307	14.63	0	0.29533	0.386724
4	0.423072	0.021537	19.64	0	0.380845	0.465299
5	0.471592	0.02152	21.91	0	0.429399	0.513785
SES						
2	0.000956	0.00877	0.11	0.913	-0.01624	0.018151
3	-0.0571	0.010931	-5.22	0	-0.07853	-0.03566
4	-0.11688	0.013043	-8.96	0	-0.14245	-0.09131
5	-0.20209	0.016012	-12.62	0	-0.23349	-0.1707
Sex	0.187266	0.002225	84.16	0	0.182904	0.191629
Age						
18-34 years	0.081909	0.003734	21.94	0	0.074589	0.08923
Year						
2016	0.025036	0.003323	7.53	0	0.018521	0.031551
2017	0.032334	0.003648	8.86	0	0.025181	0.039487
2018	0.039792	0.003824	10.41	0	0.032295	0.047289
2019	0.058794	0.003989	14.74	0	0.050974	0.066614
2020	0.031713	0.004321	7.34	0	0.023241	0.040186
age#year						
18-34 years#2016	-0.00597	0.004361	-1.37	0.171	-0.01452	0.002576
18-34 years#2017	-0.00455	0.004576	-1	0.32	-0.01353	0.004418
18-34 years#2018	0.009354	0.004563	2.05	0.04	0.000408	0.0183
18-34 years#2019	0.026177	0.004498	5.82	0	0.017358	0.034995
18-34 years#2020	0.040718	0.004646	8.76	0	0.031609	0.049827
Constant	-2.59074	0.021764	-119.04	0	-2.63341	-2.54807

Regression output for the linear regression of mental healthcare costs and age weighted for the number of insured years per unit of analysis and accounting for urbanicity, sex, year, SES, the interactions between age and year, and for clustering by 4-number postal code.

S8 Predicted costs by agegroup for BASIC-MHC costs

	Age 18-35	Age 35-65
	Marginal estimate (95%-CI)	
2015	-1.94 (-1.95; -1.93)	-2.02 (-2.03; -2.01)
2016	-1.92 (-1.93; -1.91)	-1.99 (-2.00; -1.98)
2017	-1.91 (-1.92; -1.90)	-1.99 (-2.00; -1.98)
2018	-1.89 (-1.90; -1.88)	-1.98 (-1.99; -1.97)
2019	-1.85 (-1.86; -1.84)	-1.96 (-1.97; -1.96)
2020	-1.86 (-1.87; -1.85)	-1.99 (-2.00; -1.98)

Marginal estimates for mental healthcare costs by agegroup per year. As plotted in figure 1.

S9 Marginal estimates for the association between BASIC-MHC costs and young age

	Marginal estimate (95%-CI)
2015	0.08 (0.07; 0.09)
2016	0.08 (0.07; 0.08)
2017	0.08 (0.07; 0.08)
2018	0.09 (0.08; 0.10)
2019	0.11 (0.10; 0.12)
2020	0.12 (0.12; 0.13)

Marginal estimates for the association between mental healthcare costs and age per year. As plotted in figure 2.

S10 Regression output for GP-MHC costs and age

	Coefficient	Robust std. err.	t	P>|t|	[95% conf. interval]	
Sex	0.445508	0.009777	45.57	0	0.426339	0.464677
Age						
18-34 years	-0.00075	0.012129	-0.06	0.951	-0.02453	0.023035
Year						
2016	0.068718	0.004802	14.31	0	0.059303	0.078132
2017	0.124659	0.006399	19.48	0	0.112113	0.137205
2018	0.14068	0.008349	16.85	0	0.124311	0.157049
2019	0.188638	0.009982	18.9	0	0.169068	0.208208
2020	0.208906	0.009516	21.95	0	0.190249	0.227563
age#year						
18-34 years#2016	0.0026	0.005934	0.44	0.661	-0.00903	0.014234
18-34 years#2017	0.020325	0.006377	3.19	0.001	0.007823	0.032827
18-34 years#2018	0.051625	0.006482	7.96	0	0.038917	0.064332
18-34 years#2019	0.081751	0.00659	12.41	0	0.068832	0.09467
18-34 years#2020	0.118475	0.005601	21.15	0	0.107494	0.129456
Constant	-1.93035	0.026685	-72.34	0	-1.98267	-1.87803

Regression output for the linear regression of mental healthcare costs and age weighted for the number of insured years per unit of analysis and accounting for sex, year, the interactions between age and year, and for clustering by 4-number postal code.

	Coefficient	Robust std. err.	t	P>|t|	[95% conf. interval]	
Urbanicity						
2	0.092119	0.037615	2.45	0.014	0.018371	0.165868
3	0.110258	0.042979	2.57	0.01	0.025993	0.194523
4	0.197827	0.037111	5.33	0	0.125066	0.270589
5	0.144986	0.038889	3.73	0	0.06874	0.221232
SES						
2	-0.0963	0.030795	-3.13	0.002	-0.15668	-0.03593
3	-0.14291	0.031358	-4.56	0	-0.20439	-0.08143
4	-0.20906	0.034854	-6	0	-0.2774	-0.14073
5	-0.27443	0.034085	-8.05	0	-0.34126	-0.20761
Sex	0.435036	0.002899	150.09	0	0.429354	0.440719
Age						
18-34 years	-0.0163	0.005088	-3.2	0.001	-0.02627	-0.00632
Year						
2016	0.066949	0.004769	14.04	0	0.057599	0.076299
2017	0.122878	0.006063	20.27	0	0.110991	0.134764
2018	0.141504	0.007755	18.25	0	0.1263	0.156708
2019	0.197319	0.008765	22.51	0	0.180135	0.214503
2020	0.219423	0.008822	24.87	0	0.202127	0.236719
age#year						
18-34 years#2016	0.007905	0.004595	1.72	0.085	-0.0011	0.016914
18-34 years#2017	0.027615	0.00482	5.73	0	0.018165	0.037065
18-34 years#2018	0.05952	0.005032	11.83	0	0.049653	0.069387
18-34 years#2019	0.090067	0.005384	16.73	0	0.079511	0.100624
18-34 years#2020	0.124259	0.005507	22.56	0	0.113462	0.135056
Constant	-1.91357	0.037784	-50.65	0	-1.98765	-1.83949

Regression output for the linear regression of mental healthcare costs and age weighted for the number of insured years per unit of analysis and accounting for urbanicity, sex, year, SES, the interactions between age and year, and for clustering by 4-number postal code.

S11 Predicted costs by agegroup for GP-MHC costs

	Age 18-35	Age 35-65
	Marginal estimate (95%-CI)	
2015	-1.26 (-1.28; -1.24)	-1.24 (-1.26; -1.22)
2016	-1.18 (-1.20; -1.16)	-1.17 (-1.20; -1.15)
2017	-1.11 (-1.13; -1.09)	-1.12 (-1.14; -1.10)
2018	-1.06 (-1.08; -1.03)	-1.10 (-1.12; -1.08)
2019	-0.97 (-0.99; -0.95)	-1.04 (-1.07; -1.02)
2020	-0.91 (-0.94; -0.89)	-1.02 (-1.05; -1.00)

Marginal estimates for mental healthcare costs by agegroup per year. As plotted in figure 1.

S12 Marginal estimates for the association between GP-MHC costs and young age

	Marginal estimate (95%-CI)
2015	-0.02 (-0.03; -0.01)
2016	-0.01 (-0.02; -0.00)
2017	0.01 (0.00; 0.02)
2018	0.04 (0.03; 0.05)
2019	0.07 (0.06; 0.08)
2020	0.11 (0.10; 0.12)

Marginal estimates for the association between mental healthcare costs and age per year. As plotted in figure 2.

S13 Regression output for total mental healthcare costs and sex

	Coefficient	Robust std. err.	t	P>|t|	[95% conf. interval]	
Age	0.362005	0.022842	15.85	0	0.317222	0.406788
Year						
2016	0.021327	0.003614	5.9	0	0.014241	0.028412
2017	0.04857	0.006447	7.53	0	0.03593	0.061211
2018	0.100277	0.009133	10.98	0	0.082372	0.118182
2019	0.145885	0.011058	13.19	0	0.124206	0.167564
2020	0.190424	0.008324	22.88	0	0.174104	0.206744
Sex						
Female	0.291954	0.009902	29.48	0	0.27254	0.311368
year#sex						
2016#female	0.026381	0.004336	6.08	0	0.01788	0.034882
2017#female	0.069663	0.005463	12.75	0	0.058951	0.080374
2018#female	0.112556	0.006029	18.67	0	0.100736	0.124376
2019#female	0.147313	0.006381	23.09	0	0.134804	0.159823
2020#female	0.162305	0.005642	28.77	0	0.151244	0.173365
constant	-205.523	0.018884	-11000	0	-205.56	-205.486

Regression output for the linear regression of mental healthcare costs and sex weighted for the number of insured years per unit of analysis and accounting for age, year, the interactions between sex and year, and for clustering by 4-number postal code.

	Coefficient	Robust std. err.	t	P>|t|	[95% conf. interval]	
Urbanicity						
2	0.124413	0.01569	7.93	0	0.093652	0.155175
3	0.254361	0.018253	13.94	0	0.218574	0.290148
4	0.397616	0.01625	24.47	0	0.365755	0.429477
5	0.521777	0.020758	25.14	0	0.481079	0.562474
SES						
2	-0.18827	0.016619	-11.33	0	-0.22086	-0.15569
3	-0.35527	0.017252	-20.59	0	-0.3891	-0.32145
4	-0.44693	0.017168	-26.03	0	-0.48059	-0.41327
5	-0.55132	0.018153	-30.37	0	-0.58691	-0.51572
Age	0.315804	0.006502	48.57	0	0.303057	0.328552
Year						
2016	0.017495	0.00328	5.33	0	0.011064	0.023927
2017	0.044026	0.003933	11.19	0	0.036315	0.051738
2018	0.102905	0.004399	23.39	0	0.09428	0.11153
2019	0.165694	0.005004	33.11	0	0.155883	0.175506
2020	0.208243	0.00523	39.82	0	0.197989	0.218498
Sex						
Female	0.280312	0.004703	59.6	0	0.27109	0.289533
year#sex						
2016#female	0.026197	0.004362	6.01	0	0.017646	0.034749
2017#female	0.067683	0.004954	13.66	0	0.057971	0.077395
2018#female	0.109927	0.005243	20.97	0	0.099647	0.120206
2019#female	0.144517	0.005489	26.33	0	0.133756	0.155278
2020#female	0.162309	0.005642	28.77	0	0.151248	0.173371
Constant	-205.556	0.018559	-11000	0	-205.592	-205.519

Regression output for the linear regression of mental healthcare costs and sex weighted for the number of insured years per unit of analysis and accounting for urbanicity, age, year, SES, the interactions between sex and year, and for clustering by 4-number postal code.

S14 Predicted costs by sex for total mental healthcare costs

	Male	Female
	Marginal estimate (95%-CI)	
2015	-205.37 (-205.39; -205.36)	-205.09 (-205.11; -205.08)
2016	-205.36 (-205.37; -205.34)	-205.05 (-205.06; -205.04)
2017	-205.33 (-205.34; -205.32)	-204.98 (-204.99; -204.97)
2018	-205.27 (-205.28; -205.25)	-204.88 (-204.89; -204.87)
2019	-205.21 (-205.22; -205.19)	-204.78 (-204.80; -204.77)
2020	-205.17 (-205.18; -205.15)	-204.72 (-204.74; -204.71)

Marginal estimates for mental healthcare costs by sex per year. As plotted in figure 3.

S15 Regression output for SPECIALIST-MHC costs and sex

	Coefficient	Robust std. err.	t	P>|t|	[95% conf. interval]	
Age	0.364843	0.021724	16.79	0	0.322251	0.407435
Year						
2016	0.015195	0.003743	4.06	0	0.007858	0.022532
2017	0.040469	0.006236	6.49	0	0.028242	0.052696
2018	0.091657	0.008689	10.55	0	0.074622	0.108693
2019	0.134946	0.010496	12.86	0	0.114368	0.155524
2020	0.184321	0.008255	22.33	0	0.168136	0.200506
Sex						
Female	0.244953	0.009584	25.56	0	0.226163	0.263743
year#sex						
2016#female	0.025393	0.004692	5.41	0	0.016194	0.034591
2017#female	0.066119	0.005777	11.45	0	0.054793	0.077444
2018#female	0.111907	0.006295	17.78	0	0.099566	0.124248
2019#female	0.151328	0.006626	22.84	0	0.138337	0.164319
2020#female	0.172795	0.006137	28.16	0	0.160763	0.184827
constant	-198.431	0.018078	-11000	0	-198.466	-198.396

Regression output for the linear regression of mental healthcare costs and sex weighted for the number of insured years per unit of analysis and accounting for age, year, the interactions between sex and year, and for clustering by 4-number postal code.

	Coefficient	Robust std. err.	t	P>|t|	[95% conf. interval]	
Urbanicity						
2	0.128602	0.016384	7.85	0	0.096479	0.160725
3	0.2564	0.019008	13.49	0	0.219132	0.293668
4	0.403568	0.017015	23.72	0	0.370209	0.436928
5	0.539257	0.021861	24.67	0	0.496396	0.582118
SES						
2	-0.20482	0.017626	-11.62	0	-0.23938	-0.17026
3	-0.3801	0.018172	-20.92	0	-0.41573	-0.34448
4	-0.47251	0.018141	-26.05	0	-0.50807	-0.43694
5	-0.5875	0.018685	-31.44	0	-0.62413	-0.55087
Age	0.313655	0.006887	45.54	0	0.300151	0.327158
Year						
2016	0.010916	0.003489	3.13	0.002	0.004075	0.017757
2017	0.034854	0.004213	8.27	0	0.026593	0.043114
2018	0.093234	0.00474	19.67	0	0.08394	0.102528
2019	0.154271	0.005375	28.7	0	0.143732	0.16481
2020	0.202335	0.00565	35.81	0	0.191257	0.213413
Sex						
Female	0.233886	0.005066	46.17	0	0.223954	0.243819
year#female						
2016#female	0.025242	0.004723	5.34	0	0.015982	0.034501
2017#female	0.064439	0.005413	11.9	0	0.053826	0.075052
2018#female	0.109657	0.005754	19.06	0	0.098376	0.120939
2019#female	0.148958	0.006	24.82	0	0.137194	0.160723
2020#female	0.173204	0.006195	27.96	0	0.161059	0.18535
Constant	-198.454	0.019641	-10000	0	-198.493	-198.416

Regression output for the linear regression of mental healthcare costs and sex weighted for the number of insured years per unit of analysis and accounting for urbanicity, age, year, SES, the interactions between sex and year, and for clustering by 4-number postal code.

S16 Predicted costs by sex for SPECIALIST-MHC costs

	Male	Female
	Marginal estimate (95%-CI)	
2015	-198.28 (-198.29;-198.27)	-198.05 (-198.06; -198.04)
2016	-198.27 (-198.28 ;-198.26)	-198.01 (-198.02; -198.00)
2017	-198.25 (-198.26;-198.24)	-197.95 (-197.96; -197.94)
2018	-198.19 (-198.20; -198.18)	-197.85 (-197.86; -197.83)
2019	-198.13 (-198.14; -198.11)	-197.75 (-197.76; -197.73)
2020	-198.08 (-198.09; -198.07)	-197.67 (-197.69; -197.66)

Marginal estimates for mental healthcare costs by sex per year. As plotted in figure 3.

S17 Regression output for BASIC-MHC costs and sex

	Coefficient	Robust std. err.	t	P>|t|	[95% conf. interval]	
Age	0.118508	0.007019	16.88	0	0.104746	0.13227
Year						
2016	0.025554	0.003791	6.74	0	0.018122	0.032985
2017	0.028237	0.004385	6.44	0	0.019641	0.036834
2018	0.030921	0.005132	6.03	0	0.020859	0.040983
2019	0.046037	0.005126	8.98	0	0.035987	0.056087
2020	0.02758	0.004898	5.63	0	0.017977	0.037183
Sex						
Female	0.179327	0.003779	47.46	0	0.171919	0.186736
year#sex						
2016#female	-0.00061	0.0043	-0.14	0.887	-0.00904	0.007821
2017#female	0.010304	0.004428	2.33	0.02	0.001624	0.018985
2018#female	0.023587	0.004781	4.93	0	0.014213	0.032961
2019#female	0.018499	0.004648	3.98	0	0.009386	0.027612
2020#female	0.02121	0.004681	4.53	0	0.012032	0.030388
constant	-2.12318	0.006454	-329	0	-2.13584	-2.11053

Regression output for the linear regression of mental healthcare costs and sex weighted for the number of insured years per unit of analysis and accounting for age, year, the interactions between sex and year, and for clustering by 4-number postal code.

	Coefficient	Robust std. err.	t	P>|t|	[95% conf. interval]	
Urbanicity						
2	0.195861	0.022902	8.55	0	0.150958	0.240763
3	0.341085	0.023308	14.63	0	0.295386	0.386783
4	0.423104	0.021539	19.64	0	0.380875	0.465333
5	0.471618	0.021522	21.91	0	0.429422	0.513814
SES						
2	0.001139	0.008769	0.13	0.897	-0.01605	0.018332
3	-0.05694	0.010929	-5.21	0	-0.07837	-0.03551
4	-0.11671	0.013043	-8.95	0	-0.14229	-0.09114
5	-0.202	0.016014	-12.61	0	-0.2334	-0.1706
Age	0.093141	0.002207	42.21	0	0.088814	0.097467
Year						
2016	0.023115	0.003897	5.93	0	0.015475	0.030755
2017	0.025921	0.004296	6.03	0	0.017498	0.034344
2018	0.031846	0.004473	7.12	0	0.023077	0.040616
2019	0.058879	0.004668	12.61	0	0.049726	0.068031
2020	0.035119	0.004881	7.2	0	0.025551	0.044688
Sex						
Female	0.17548	0.003626	48.39	0	0.16837	0.182589
year#sex						
2016#female	-0.00019	0.004363	-0.04	0.965	-0.00874	0.008363
2017#female	0.009643	0.004456	2.16	0.031	0.000907	0.018379
2018#female	0.022077	0.004581	4.82	0	0.013095	0.031058
2019#female	0.017597	0.004604	3.82	0	0.008571	0.026624
2020#female	0.021163	0.004716	4.49	0	0.011916	0.030409
constant	-2.40146	0.021725	-110.54	0	-2.44405	-2.35886

Regression output for the linear regression of mental healthcare costs and sex weighted for the number of insured years per unit of analysis and accounting for urbanicity, age, year, SES, the interactions between sex and year, and for clustering by 4-number postal code.

S18 Predicted costs by sex for BASIC-MHC costs

	Male	Female
	Marginal estimate (95%-CI)	
2015	-2.08 (-2.09;-2.07)	-1.90 (-1.91;-0.89)
2016	-2.05 (-2.06;-2.04)	-1.88 (-1.89;-1.87)
2017	-2.05 (-12.06; -2.04)	-1.87 (-1.88; -1.86)
2018	-2.05 (-2.06;-2.04)	-1.85 (-1.86;-1.84)
2019	-2.02 (-2.03; -2.01)	-1.83 (-1.83;-1.82)
2020	-2.04 (-2.05;-2.03)	-1.85 (-1.85;-1.84)

Marginal estimates for mental healthcare costs by sex per year. As plotted in figure 3.

S19 Regression output for GP-MHC costs and sex

	Coefficient	Robust std. err.	t	P>|t|	[95% conf. interval]	
Age	0.04573	0.014515	3.15	0.002	0.017272	0.074188
Year						
2016	0.063828	0.004815	13.25	0	0.054387	0.073269
2017	0.121777	0.007253	16.79	0	0.107557	0.135998
2018	0.150029	0.009402	15.96	0	0.131596	0.168463
2019	0.213018	0.011595	18.37	0	0.190285	0.235752
2020	0.248955	0.009589	25.96	0	0.230156	0.267754
Sex						
Female	0.436392	0.008152	53.53	0	0.42041	0.452375
year#sex						
2016#female	0.011386	0.003982	2.86	0.004	0.00358	0.019193
2017#female	0.019156	0.004795	3.99	0	0.009755	0.028558
2018#female	0.015977	0.005509	2.9	0.004	0.005177	0.026777
2019#female	0.00679	0.006132	1.11	0.268	-0.00523	0.018814
2020#female	0.001239	0.005049	0.25	0.806	-0.00866	0.011137
constant	-1.49583	0.01707	-87.63	0	-1.5293	-1.46237

Regression output for the linear regression of mental healthcare costs and sex weighted for the number of insured years per unit of analysis and accounting for age, year, the interactions between sex and year, and for clustering by 4-number postal code.

	Coefficient	Robust std. err.	t	P>|t|	[95% conf. interval]	
Urbanicity						
2	0.092288	0.037614	2.45	0.014	0.018541	0.166035
3	0.110395	0.04298	2.57	0.01	0.026128	0.194663
4	0.197889	0.037112	5.33	0	0.125127	0.270651
5	0.145064	0.038886	3.73	0	0.068822	0.221305
SES						
2	-0.09582	0.030794	-3.11	0.002	-0.15619	-0.03544
3	-0.14247	0.031356	-4.54	0	-0.20394	-0.08099
4	-0.20856	0.034855	-5.98	0	-0.2769	-0.14022
5	-0.27413	0.034084	-8.04	0	-0.34096	-0.20731
Age	0.036009	0.003859	9.33	0	0.028444	0.043575
Year						
2016	0.064234	0.004543	14.14	0	0.055327	0.073142
2017	0.123513	0.005678	21.75	0	0.112381	0.134645
2018	0.15515	0.007212	21.51	0	0.14101	0.16929
2019	0.226533	0.008157	27.77	0	0.21054	0.242525
2020	0.262323	0.008166	32.12	0	0.246313	0.278333
Sex						
Female	0.428185	0.00407	105.2	0	0.420205	0.436165
year#sex						
2016#female	0.010515	0.003914	2.69	0.007	0.002842	0.018188
2017#female	0.016829	0.004287	3.93	0	0.008423	0.025235
2018#female	0.012372	0.0044	2.81	0.005	0.003745	0.020998
2019#female	0.002342	0.004392	0.53	0.594	-0.00627	0.010953
2020#female	-0.00103	0.004513	-0.23	0.819	-0.00988	0.007813
Constant	-1.49288	0.037664	-39.64	0	-1.56672	-1.41903

Regression output for the linear regression of mental healthcare costs and sex weighted for the number of insured years per unit of analysis and accounting for urbanicity, age, year, SES, the interactions between sex and year, and for clustering by 4-number postal code.

S20 Predicted costs by sex for GP-MHC costs

	Male	Female
	Marginal estimate (95%-CI)	
2015	-1.46 (-1.48; -1.44)	-1.03 (-1.05; -1.01)
2016	-1.39 (-1.42; -1.38)	-0.96 (-0.98; -0.93)
2017	-1.34 (-1.36; -1.32)	-0.89 (-0.92; -0.87)
2018	-1.31 (-1.33; -1.28)	-0.87 (-0.89; -0.84)
2019	-1.23 (-1.26; -1.21)	-0.80 (-0.83; -0.78)
2020	-1.20 (-1.22; -1.17)	-0.77 (-0.80; -0.74)

Marginal estimates for mental healthcare costs by sex per year. As plotted in figure 3.

S21 Regression output for total mental healthcare costs, age and sex

	Coefficient	Robust std. err.	t	P>|t|	[95% conf. interval]	
Age						
18-34 years	0.14382	0.020586	6.99	0	0.10346	0.184179
Year						
2016	0.020925	0.003828	5.47	0	0.01342	0.028431
2017	0.046111	0.005475	8.42	0	0.035376	0.056845
2018	0.087869	0.00758	11.59	0	0.073009	0.102729
2019	0.115373	0.009795	11.78	0	0.096169	0.134577
2020	0.145912	0.008674	16.82	0	0.128905	0.162919
age#year						
18-34 years#2016	0.002902	0.00726	0.4	0.689	-0.01133	0.017135
18-34 years#2017	0.011405	0.009213	1.24	0.216	-0.00666	0.029468
18-34 years#2018	0.042389	0.010548	4.02	0	0.021709	0.063069
18-34 years#2019	0.095883	0.010361	9.25	0	0.07557	0.116196
18-34 years#2020	0.136325	0.00926	14.72	0	0.118171	0.15448
Sex						
Female	0.196852	0.009493	20.74	0	0.178241	0.215464
age#sex						
18-34 years#female	0.283615	0.009197	30.84	0	0.265583	0.301647
year#sex						
2016#female	0.023192	0.005159	4.5	0	0.013078	0.033306
2017#female	0.051669	0.005887	8.78	0	0.040128	0.06321
2018#female	0.083501	0.006423	13	0	0.070909	0.096094
2019#female	0.113074	0.007156	15.8	0	0.099045	0.127103
2020#female	0.116902	0.006628	17.64	0	0.103906	0.129897
age#year#sex						
18-34 years#2016#female	0.007572	0.009563	0.79	0.429	-0.01118	0.026321
18-34 years#2017#female	0.048588	0.01051	4.62	0	0.027981	0.069194
18-34 years#2018#female	0.078258	0.011119	7.04	0	0.056458	0.100059
18-34 years#2019#female	0.090746	0.011541	7.86	0	0.06812	0.113373
18-34 years#2020#female	0.121252	0.011844	10.24	0	0.098031	0.144474
constant	-205.45	0.019797	-10000	0	-205.489	-205.411

Regression output for the linear regression of mental healthcare costs, age, and sex weighted for the number of insured years per unit of analysis and accounting for year, the interactions between age, sex and year, and for clustering by 4-number postal code.

	Coefficient	Robust std. err.	t	P>|t|	[95% conf. interval]	
Urbanicity						
2	0.123977	0.015682	7.91	0	0.093231	0.154723
3	0.253889	0.01825	13.91	0	0.218106	0.289671
4	0.396776	0.016231	24.45	0	0.364953	0.428598
5	0.519508	0.020674	25.13	0	0.478975	0.560041
SES						
2	-0.18882	0.016544	-11.41	0	-0.22126	-0.15639
3	-0.35548	0.017192	-20.68	0	-0.38919	-0.32178
4	-0.44712	0.017123	-26.11	0	-0.48069	-0.41354
5	-0.55123	0.018104	-30.45	0	-0.58673	-0.51574
Age						
18-34 years	0.095419	0.009616	9.92	0	0.076565	0.114273
Year						
2016	0.01607	0.003764	4.27	0	0.00869	0.02345
2017	0.038102	0.004415	8.63	0	0.029447	0.046758
2018	0.08627	0.004943	17.45	0	0.076578	0.095961
2019	0.129891	0.005409	24.02	0	0.119287	0.140495
2020	0.160669	0.005761	27.89	0	0.149374	0.171965
age#year						
18-34 years#2016	0.005858	0.007232	0.81	0.418	-0.00832	0.020037
18-34 years#2017	0.021399	0.007933	2.7	0.007	0.005845	0.036954
18-34 years#2018	0.054841	0.008534	6.43	0	0.03811	0.071573
18-34 years#2019	0.111703	0.009039	12.36	0	0.093981	0.129425
18-34 years#2020	0.146311	0.009324	15.69	0	0.12803	0.164592
Sex						
Female	0.191591	0.005666	33.81	0	0.180482	0.202701
age#sex						
18-34 years#female	0.266184	0.009196	28.95	0	0.248154	0.284214
year#sex						
2016#female	0.022457	0.005185	4.33	0	0.01229	0.032624
2017#female	0.049788	0.005724	8.7	0	0.038565	0.061011
2018#female	0.080457	0.006042	13.32	0	0.068611	0.092303
2019#female	0.108746	0.006369	17.07	0	0.096259	0.121233
2020#female	0.115034	0.006577	17.49	0	0.102139	0.127929
age#year#sex						
18-34 years#2016#female	0.009598	0.00974	0.99	0.324	-0.0095	0.028694
18-34 years#2017#female	0.049327	0.010611	4.65	0	0.028523	0.070131
18-34 years#2018#female	0.0807	0.01129	7.15	0	0.058565	0.102835
18-34 years#2019#female	0.096408	0.011762	8.2	0	0.073348	0.119469
18-34 years#2020#female	0.127246	0.012039	10.57	0	0.103641	0.15085
constant	-205.481	0.018658	-11000	0	-205.517	-205.444

Regression output for the linear regression of mental healthcare costs, age, and sex weighted for the number of insured years per unit of analysis and accounting for urbanicity, year, SES, the interactions between age, sex and year, and for clustering by 4-number postal code.

S22 Marginal estimates for the association between age and total mental healthcare costs by sex

	Male	Female
	Marginal estimate (95%-CI)	
2015	0.10 (0.08; 0.11)	0.36 (0.34; 0.38)
2016	0.10 (0.08; 0.12)	0.38 (0.36; 0.39)
2017	0.12 (0.10; 0.14)	0.43 (0.42; 0.45)
2018	0.15 (0.13; 0.17)	0.50 (0.48; 0.51)
2019	0.21 (0.19; 0.23)	0.57 (0.55; 0.59)
2020	0.24 (0.22; 0.26)	0.64 (0.62; 0.65)

Marginal estimates for the association between mental healthcare costs and age by sex and per year. As plotted in figure 4.

S23 Regression output for SPECIALIST-MHC costs, age and sex

	Coefficient	Robust std. err.	t	P>|t|	[95% conf. interval]	
Age						
18-34 years	0.145019	0.019565	7.41	0	0.106661	0.183377
Year						
2016	0.014273	0.004048	3.53	0	0.006336	0.02221
2017	0.03729	0.005506	6.77	0	0.026495	0.048084
2018	0.07949	0.007428	10.7	0	0.064927	0.094053
2019	0.105298	0.009414	11.18	0	0.086841	0.123756
2020	0.140562	0.008607	16.33	0	0.123687	0.157437
age#year						
18-34 years#2016	0.00445	0.007674	0.58	0.562	-0.0106	0.019496
18-34 years#2017	0.013535	0.009504	1.42	0.154	-0.0051	0.032168
18-34 years#2018	0.041745	0.010721	3.89	0	0.020726	0.062763
18-34 years#2019	0.093482	0.010787	8.67	0	0.072333	0.114631
18-34 years#2020	0.134269	0.009875	13.6	0	0.114909	0.153629
Sex						
Female	0.151357	0.009366	16.16	0	0.132994	0.169721
age#sex						
18-34 years#female	0.279054	0.009912	28.15	0	0.25962	0.298488
year#sex						
2016#female	0.021908	0.005559	3.94	0	0.011008	0.032807
2017#female	0.047578	0.006367	7.47	0	0.035095	0.060061
2018#female	0.079855	0.00687	11.62	0	0.066386	0.093324
2019#female	0.111485	0.007555	14.76	0	0.096672	0.126298
2020#female	0.119948	0.00721	16.64	0	0.105811	0.134085
age#year#sex						
18-34 years#2016#female	0.008501	0.010414	0.82	0.414	-0.01192	0.028918
18-34 years#2017#female	0.050306	0.011493	4.38	0	0.027773	0.072839
18-34 years#2018#female	0.087162	0.012149	7.17	0	0.063344	0.110981
18-34 years#2019#female	0.107162	0.012741	8.41	0	0.082183	0.132142
18-34 years#2020#female	0.142918	0.013097	10.91	0	0.11724	0.168596
constant	-198.357	0.019083	-10000	0	-198.394	-198.32

Regression output for the linear regression of mental healthcare costs, age, and sex weighted for the number of insured years per unit of analysis and accounting for year, the interactions between age, sex and year, and for clustering by 4-number postal code.

	Coefficient	Robust std. err.	t	P>|t|	[95% conf. interval]	
Urbanicity						
2	0.128154	0.016374	7.83	0	0.096051	0.160258
3	0.255917	0.019004	13.47	0	0.218657	0.293176
4	0.402718	0.016993	23.7	0	0.369401	0.436036
5	0.536961	0.021776	24.66	0	0.494266	0.579655
SES						
2	-0.20541	0.01755	-11.7	0	-0.23982	-0.171
3	-0.38035	0.018111	-21	0	-0.41585	-0.34484
4	-0.47273	0.018096	-26.12	0	-0.50821	-0.43725
5	-0.58743	0.018637	-31.52	0	-0.62397	-0.55089
Age						
18-34 years	0.091457	0.010221	8.95	0	0.071417	0.111496
Year						
2016	0.00905	0.004004	2.26	0.024	0.001199	0.016901
2017	0.028256	0.004729	5.98	0	0.018985	0.037527
2018	0.076885	0.005325	14.44	0	0.066444	0.087325
2019	0.119208	0.005842	20.4	0	0.107754	0.130663
2020	0.15523	0.006202	25.03	0	0.143071	0.16739
age#year						
18-34 years#2016	0.007183	0.007724	0.93	0.352	-0.00796	0.022326
18-34 years#2017	0.023413	0.008477	2.76	0.006	0.006793	0.040033
18-34 years#2018	0.054062	0.009125	5.92	0	0.036171	0.071953
18-34 years#2019	0.109646	0.009671	11.34	0	0.090685	0.128607
18-34 years#2020	0.145063	0.009946	14.58	0	0.125562	0.164564
Sex						
Female	0.146819	0.006063	24.22	0	0.134932	0.158706
age#sex						
18-34 years#female	0.261152	0.009983	26.16	0	0.24158	0.280724
year#sex						
2016#female	0.021126	0.00558	3.79	0	0.010187	0.032065
2017#female	0.045861	0.006269	7.32	0	0.033571	0.058151
2018#female	0.076939	0.006592	11.67	0	0.064015	0.089863
2019#female	0.107306	0.006936	15.47	0	0.093706	0.120905
2020#female	0.118205	0.007194	16.43	0	0.1041	0.132309
age#year#sex						
18-34 years#2016#female	0.010767	0.010593	1.02	0.31	-0.01	0.031537
18-34 years#2017#female	0.05146	0.011655	4.42	0	0.02861	0.074311
18-34 years#2018#female	0.090406	0.012399	7.29	0	0.066097	0.114716
18-34 years#2019#female	0.113751	0.012946	8.79	0	0.088369	0.139133
18-34 years#2020#female	0.149845	0.013261	11.3	0	0.123845	0.175845
Constant	-198.379	0.01974	-10000	0	-198.417	-198.34

Regression output for the linear regression of mental healthcare costs, age, and sex weighted for the number of insured years per unit of analysis and accounting for urbanicity, year, SES, the interactions between age, sex and year, and for clustering by 4-number postal code.

S24 Marginal estimates for the association between age and SPECIALIST-MHC costs by sex

	Male	Female
	Marginal estimate (95%-CI)	
2015	0.09 (0.07; 0.11)	0.35 (0.33; 0.37)
2016	0.10 (0.08; 0.12)	0.37 (0.35; 0.39)
2017	0.11 (0.10; 0.13)	0.43 (0.41; 0.44)
2018	0.15 (0.13; 0.16)	0.50 (0.48; 0.51)
2019	0.20 (0.18; 0.22)	0.58 (0.56; 0.59)
2020	0.24 (0.22; 0.26)	0.65 (0.63; 0.67)

Marginal estimates for the association between mental healthcare costs and age by sex and per year. As plotted in figure 4.

S25 Regression output for BASIC-MHC costs, age and sex

	Coefficient	Robust std. err.	t	P>|t|	[95% conf. interval]	
Age						
18-34 years	0.078346	0.009257	8.46	0	0.060197	0.096495
Year						
2016	0.028079	0.004806	5.84	0	0.018657	0.037501
2017	0.034684	0.005301	6.54	0	0.024291	0.045076
2018	0.030836	0.005638	5.47	0	0.019782	0.04189
2019	0.037174	0.005867	6.34	0	0.025672	0.048676
2020	0.013353	0.005913	2.26	0.024	0.00176	0.024945
age#year						
18-34 years#2016	-0.00713	0.007068	-1.01	0.313	-0.02099	0.006726
18-34 years#2017	-0.01801	0.007453	-2.42	0.016	-0.03262	-0.0034
18-34 years#2018	0.001463	0.008103	0.18	0.857	-0.01442	0.017349
18-34 years#2019	0.026911	0.007258	3.71	0	0.012681	0.041141
18-34 years#2020	0.042198	0.007332	5.76	0	0.027823	0.056572
Sex						
Female	0.157675	0.00457	34.5	0	0.148715	0.166635
age#sex						
18-34 years#female	0.064702	0.006884	9.4	0	0.051206	0.078197
year#sex						
2016#female	-0.00069	0.005723	-0.12	0.904	-0.01191	0.010533
2017#female	0.002637	0.00579	0.46	0.649	-0.00871	0.013988
2018#female	0.020938	0.006045	3.46	0.001	0.009086	0.03279
2019#female	0.022297	0.00602	3.7	0	0.010494	0.034099
2020#female	0.023945	0.006081	3.94	0	0.012023	0.035867
age#year#sex						
18-34 years#2016#female	-0.00032	0.008411	-0.04	0.97	-0.01681	0.01617
18-34 years#2017#female	0.021328	0.008627	2.47	0.013	0.004414	0.038241
18-34 years#2018#female	0.006037	0.009105	0.66	0.507	-0.01182	0.023889
18-34 years#2019#female	-0.01294	0.00851	-1.52	0.128	-0.02963	0.003739
18-34 years#2020#female	-0.00992	0.008538	-1.16	0.245	-0.02666	0.006821
constant	-2.10967	0.006721	-313.87	0	-2.12285	-2.0965

Regression output for the linear regression of mental healthcare costs, age, and sex weighted for the number of insured years per unit of analysis and accounting for year, the interactions between age, sex and year, and for clustering by 4-number postal code.

	Coefficient	Robust std. err.	t	P>|t|	[95% conf. interval]	
Urbanicity						
2	0.195771	0.022901	8.55	0	0.150871	0.240671
3	0.340988	0.023307	14.63	0	0.295292	0.386684
4	0.422947	0.021536	19.64	0	0.380723	0.465171
5	0.47123	0.021517	21.9	0	0.429043	0.513416
SES						
2	0.001017	0.008768	0.12	0.908	-0.01617	0.018208
3	-0.057	0.010928	-5.22	0	-0.07843	-0.03557
4	-0.11676	0.013042	-8.95	0	-0.14233	-0.09119
5	-0.20199	0.01601	-12.62	0	-0.23338	-0.1706
Age						
18-34 years	0.054876	0.005764	9.52	0	0.043574	0.066177
Year						
2016	0.025096	0.004902	5.12	0	0.015485	0.034707
2017	0.031468	0.005276	5.96	0	0.021123	0.041814
2018	0.029595	0.005568	5.32	0	0.018679	0.040512
2019	0.048149	0.005622	8.56	0	0.037126	0.059172
2020	0.019698	0.005982	3.29	0.001	0.007969	0.031427
age#year						
18-34 years#2016	-0.00561	0.00721	-0.78	0.437	-0.01975	0.008529
18-34 years#2017	-0.01562	0.007651	-2.04	0.041	-0.03062	-0.00062
18-34 years#2018	0.007667	0.007519	1.02	0.308	-0.00708	0.02241
18-34 years#2019	0.032363	0.007382	4.38	0	0.01789	0.046836
18-34 years#2020	0.045802	0.007473	6.13	0	0.031151	0.060453
Sex						
Female	0.157441	0.00463	34	0	0.148363	0.16652
age#sex						
18-34 years#female	0.054198	0.006441	8.41	0	0.041569	0.066827
year#sex						
2016#female	-9.5E-05	0.00576	-0.02	0.987	-0.01139	0.011199
2017#female	0.00177	0.005834	0.3	0.762	-0.00967	0.013208
2018#female	0.020358	0.006123	3.33	0.001	0.008354	0.032362
2019#female	0.021241	0.006072	3.5	0	0.009336	0.033145
2020#female	0.023979	0.006177	3.88	0	0.011868	0.036091
age#year#sex						
18-34 years#2016#female	-0.00072	0.008535	-0.08	0.933	-0.01745	0.016018
18-34 years#2017#female	0.022386	0.008818	2.54	0.011	0.005097	0.039676
18-34 years#2018#female	0.003734	0.008804	0.42	0.671	-0.01353	0.020995
18-34 years#2019#female	-0.01214	0.008631	-1.41	0.16	-0.02906	0.004781
18-34 years#2020#female	-0.00986	0.008582	-1.15	0.251	-0.02668	0.006969
Constant	-2.38845	0.021854	-109.29	0	-2.4313	-2.3456

Regression output for the linear regression of mental healthcare costs, age, and sex weighted for the number of insured years per unit of analysis and accounting for urbanicity, year, SES, the interactions between age, sex and year, and for clustering by 4-number postal code.

S26 Marginal estimates for the association between age and BASIC-MHC costs by sex

	Male	Female
	Marginal estimate (95%-CI)	
2015	0.05 (0.04; 0.07)	0.11 (0.10; 0.12)
2016	0.05 (0.04; 0.06)	0.10 (0.10; 0.11)
2017	0.04 (0.03; 0.05)	0.12 (0.11; 0.12)
2018	0.06 (0.05; 0.07)	0.12 (0.11; 0.13)
2019	0.09 (0.08; 0.10)	0.13 (0.12; 0.14)
2020	0.10 (0.09; 0.11)	0.15 (0.14; 0.15)

Marginal estimates for the association between mental healthcare costs and age by sex and per year. As plotted in figure 4.

S27 Regression output for GP-MHC costs, age and sex

	Coefficient	Robust std. err.	t	P>|t|	[95% conf. interval]	
Age						
18-34 years	-0.04537	0.012211	-3.72	0	-0.06931	-0.02143
Year						
2016	0.061358	0.00515	11.92	0	0.051262	0.071454
2017	0.114593	0.006593	17.38	0	0.101667	0.12752
2018	0.132025	0.008642	15.28	0	0.115082	0.148967
2019	0.180842	0.010859	16.65	0	0.159552	0.202132
2020	0.203664	0.009504	21.43	0	0.18503	0.222298
age#year						
18-34 years#2016	0.008001	0.008606	0.93	0.353	-0.00887	0.024873
18-34 years#2017	0.022712	0.010251	2.22	0.027	0.002615	0.042809
18-34 years#2018	0.054667	0.009824	5.56	0	0.035406	0.073927
18-34 years#2019	0.095475	0.009106	10.48	0	0.077622	0.113328
18-34 years#2020	0.132623	0.00832	15.94	0	0.116311	0.148935
Sex						
Female	0.406162	0.008522	47.66	0	0.389454	0.422871
age#sex						
18-34 years#female	0.089857	0.006432	13.97	0	0.077247	0.102467
year#sex						
2016#female	0.014745	0.004696	3.14	0.002	0.005538	0.023953
2017#female	0.020166	0.005118	3.94	0	0.010132	0.0302
2018#female	0.017343	0.005537	3.13	0.002	0.006488	0.028198
2019#female	0.015621	0.006291	2.48	0.013	0.003287	0.027955
2020#female	0.01052	0.005523	1.9	0.057	-0.00031	0.021347
age#year#sex						
18-34 years#2016#female	-0.01066	0.008861	-1.2	0.229	-0.02804	0.006709
18-34 years#2017#female	-0.00433	0.010971	-0.39	0.693	-0.02584	0.01718
18-34 years#2018#female	-0.00563	0.010278	-0.55	0.584	-0.02578	0.01452
18-34 years#2019#female	-0.02738	0.00932	-2.94	0.003	-0.04565	-0.00911
18-34 years#2020#female	-0.02837	0.010292	-2.76	0.006	-0.04855	-0.0082
constant	-1.46519	0.017853	-82.07	0	-1.50019	-1.43019

Regression output for the linear regression of mental healthcare costs, age, and sex weighted for the number of insured years per unit of analysis and accounting for year, the interactions between age, sex and year, and for clustering by 4-number postal code.

	Coefficient	Robust std. err.	t	P>|t|	[95% conf.	interval]
Urbanicity						
2	0.092096	0.037615	2.45	0.014	0.018347	0.165844
3	0.110211	0.042979	2.56	0.01	0.025945	0.194477
4	0.19767	0.037111	5.33	0	0.124909	0.270432
5	0.144527	0.038885	3.72	0	0.068289	0.220765
SES						
2	-0.09623	0.030788	-3.13	0.002	-0.15659	-0.03587
3	-0.14278	0.031354	-4.55	0	-0.20426	-0.08131
4	-0.20891	0.034854	-5.99	0	-0.27724	-0.14057
5	-0.2743	0.034081	-8.05	0	-0.34112	-0.20748
Age						
18-34 years	-0.05891	0.006179	-9.53	0	-0.07103	-0.0468
Year						
2016	0.059448	0.005162	11.52	0	0.049327	0.06957
2017	0.112782	0.006361	17.73	0	0.100311	0.125252
2018	0.133861	0.007803	17.16	0	0.118564	0.149159
2019	0.191319	0.008796	21.75	0	0.174073	0.208565
2020	0.214664	0.008706	24.66	0	0.197594	0.231733
age#year						
18-34 years#2016	0.014906	0.006714	2.22	0.026	0.001743	0.028069
18-34 years#2017	0.033243	0.006927	4.8	0	0.019663	0.046823
18-34 years#2018	0.064648	0.007155	9.04	0	0.050619	0.078676
18-34 years#2019	0.104989	0.007182	14.62	0	0.090908	0.11907
18-34 years#2020	0.140391	0.007309	19.21	0	0.12606	0.154722
Sex						
Female	0.399581	0.004725	84.57	0	0.390318	0.408845
age#sex						
18-34 years#female	0.085526	0.006597	12.96	0	0.072591	0.098461
year#sex						
2016#female	0.014978	0.004758	3.15	0.002	0.00565	0.024307
2017#female	0.020165	0.005185	3.89	0	0.01	0.030331
2018#female	0.015284	0.005403	2.83	0.005	0.004689	0.025878
2019#female	0.012001	0.005535	2.17	0.03	0.00115	0.022853
2020#female	0.009536	0.005547	1.72	0.086	-0.00134	0.020412
age2#year#sex						
18-34 years#2016#female	-0.01388	0.008313	-1.67	0.095	-0.03018	0.002417
18-34 years#2017#female	-0.011	0.008536	-1.29	0.198	-0.02774	0.005736
18-34 years#2018#female	-0.01002	0.008755	-1.14	0.253	-0.02718	0.007148
18-34 years#2019#female	-0.02983	0.008683	-3.44	0.001	-0.04686	-0.01281
18-34 years#2020#female	-0.03229	0.008694	-3.71	0	-0.04934	-0.01525
Constant	-1.46068	0.03773	-38.71	0	-1.53465	-1.3867

Regression output for the linear regression of mental healthcare costs, age, and sex weighted for the number of insured years per unit of analysis and accounting for urbanicity, year, SES, the interactions between age, sex and year, and for clustering by 4-number postal code.

S28 Marginal estimates for the association between age and GP-MHC costs by sex

	Male	Female
	Marginal estimate (95%-CI)	
2015	-0.06 (-0.07; -0.05)	0.03 (0.01; 0.04)
2016	-0.04 (-0.06; -0.03)	0.03 (0.02; 0.04)
2017	-0.03 (-0.04; -0.01)	0.05 (0.04; 0.06)
2018	0.01 (-0.01; 0.02)	0.08 (0.07; 0.09)
2019	0.05 (0.03; 0.06)	0.10 (0.09; 0.11)
2020	0.08 (0.07; 0.09)	0.13 (0.12; 0.15)

Marginal estimates for the association between mental healthcare costs and age by sex and per year. As plotted in figure 4.
