# Supplementary material for: Mental healthcare expenditure among adults by type of mental healthcare and its association with age and sex in the Netherlands between 2015 and 2020
Source: PLoS One. 2025 Aug 25;20(8):e0330365. doi: 10.1371/journal.pone.0330365 (PMC12377576; doi:10.1371/journal.pone.0330365)
Supplement: Attachment — Submitted filename: Response to reviewers PlosONE_V2.docx [file pone.0330365.s003.docx]

**Reviewer #1:** 
The authors utilized a health insurance database linked with postal codes and data from the Central Bureau for Statistics to estimate mental health costs in the Netherlands. They report that younger people incur higher costs than the 35–65-year-old group. The handling of missing data and covariates was generally reasonable. However, I have a few comments and questions I would like to raise:

1. Given that the database provides age information subdivided into groups (18–20 years and 5-year intervals thereafter), why did the analysis use a dichotomized age variable with a cut-off at 35 years? Would using 5- or 10-year age groups allow for a more detailed understanding of age-related differences in mental healthcare costs?

*Response: We thank the reviewer for this important remark, which was also raised by reviewer 2. We chose this cut-off to align with a previous national report on the rising prevalence of common mental health conditions among young adults in the Netherlands, to allow for comparability.(1) There is also no universally agreed definition of “young adulthood”, and different studies use varying age thresholds.(2) In addition, we used the same cut-off in our previous work on mental healthcare costs in the Netherlands to ensure consistency. (3) In that earlier work, we also conducted an exploratory analysis using smaller age bands.(3) These analyses showed that mental healthcare costs were lower for individuals aged 18–19 compared to those aged 20–34, while costs within the 20-34 age range were relatively stable across the smaller intervals. We hypothesized that the observed differences -particularly between those aged 18-19 and older groups- may reflect structural features of the Dutch mental health system. Specifically, those under the age of 18 are served by different care organizations than those aged 18 and older, which can sometimes lead to discontinuities in care at this transition point.*

*As we previously conducted this analysis for total mental healthcare costs, we did not repeat it in the current paper. In response to the Reviewer’s suggestion, however, we have revised the manuscript to clarify our rationale for the chosen cut-off. We have added the following sentence to the methods section:*

*“In the absence of a universally agreed-upon definition of young adulthood, we chose this cut-off for young adulthood in line with a previous report from the Netherlands on the rise in common mental health disorders to facilitate comparability.” [lines 95-97]*

2. Were younger people already using more mental health resources before 2015, or was there a crossover in usage patterns at some point during the study period?

*Response: We thank the reviewer for this comment. Unfortunately, we did not have access to insurance data from before 2015 for this work. Comparing insurance data across longer time periods is challenging due to changes in the mental healthcare financing system. Specifically, a major reform took place in the Netherlands, in 2014, aimed at reducing costs. After this reform, only people meeting the criteria for a DSM diagnosis were eligible for reimbursed mental healthcare, while those with, milder psychological complaints were limited to a restricted number of treatment sessions. This policy change makes it difficult to directly compare mental healthcare usage before and after the reform using insurance data.*

*We have added the following to the limitations section:*

*“The lack of data on the period before 2015 meant we could not ascertain whether the overall higher mental healthcare costs in youth are a new phenomenon.” [lines 306-307]*

3. I noticed that mental health costs have increased year by year, but has the [number of visits or usage] by the public also increased annually? Or does this upward trend simply reflect increases in the cost of living or general inflation? How does this trend compare to that of other medical services?

*Response: We thank you for noting this important point. In our dataset there was no information on the number of visits. From government reports we know that the number of care trajectories (‘prestaties’ in the insurance system) did increase over the years 2017-2020. (4) It is thus likely that increased costs are in part due to increased use of mental healthcare, but inflation and increased costs of living are probably also contributing to the overall rise. Reports from the government have shown that all healthcare costs have risen in past years. (4)
In our work we have focussed mostly on the proportion of costs spend on specific demographics each year (figure 2 and 4), for which absolute costs which can vary due to inflation are not as relevant.
We have previously examined how those proportions relate to those in medical specialist care and primary care and found younger age was associated with lower general practitioner costs, but this association weakened over time.(3) Younger age was also associated with lower medical specialist costs.(3)
We have clarified the issue of inflation in the limitations section by adding the following sentence:*

*“Another issue is that we did not correct for inflation, meaning that our calculated prediction of log-transformed standardised costs (as shown in figure 1 and 3) could in part have been influenced by inflation. However, the marginal estimate of the association between younger age and costs (as shown in figure 2 and 4) is not influenced by this, as these reflect the proportion of mental healthcare costs made by younger people.” [lines 312-316]*

4. Is it possible to provide information on the possible reasons for using mental health services, such as substance abuse, alcohol-related problems, emotional issues, self-harm, or psychosis? I know the authors discussed many possible reasons in the Discussion section, but is it possible to extract such information from the database itself?

*Response: We agree with the reviewer that this would be valuable information. Unfortunately, it is not possible for us to extract this information from the postal code aggregated database we received from the insurance companies. We have clarified this in the methods by adding:*

“*There was no information on the reason for mental healthcare use in the database, as this would require individual-level data.” [lines 91-92]*

5. The authors mention in the limitation section that “[Another major limitation is that we do not have the most recent data, meaning we could not examine how the found trends developed until the present.]” This is indeed unfortunate, as the period from 2020 to 2022 saw the global impact of COVID-19, during which the mental health system likely underwent significant changes.

*Response: We fully agree with the reviewer that this is unfortunate, and we hope that we will be able to obtain the more recent data from insurers in the future. In the case of the Netherlands, the basic structure of the mental healthcare system has remained unchanged since COVID-19. However, there was a transition in the reimbursement system (from the ‘DBC’ to the ‘ZPM’ model) that may have influenced reported expenditures.(5) Since this change partly overlapped with the pandemic, it would be challenging to disentangle the effects of the reimbursement reform from those of COVID-19. For future work with more up-to-date data, it will be relevant to investigate whether this new reimbursement system has led to different cost patterns across age groups.*

**Reviewer #2:** 
This is a well-executed study using large-scale national data to explore important trends in mental healthcare use. The analysis is methodologically solid, and the findings have clear relevance for policy and planning. I have three suggestions that may help strengthen the manuscript:

1. In the Methods section, the choice to dichotomize age into 18–34 and 35–64 simplifies the analysis but may limit interpretability. This grouping could mask important differences within the broad 18–34 range, such as between individuals in early versus late young adulthood. It would strengthen the methodological transparency to explain the rationale behind this cutoff, and if data allow, to explore whether narrower age categories yield different or more detailed patterns in expenditure.

*Response: We thank the reviewer for noting this important point, which was also noted by reviewer 1. We chose this cut-off to align with a previous national report on the rising prevalence of common mental health conditions among young adults in the Netherlands, to allow for comparability.(1) There is also no universally agreed definition of “young adulthood”, and different studies use varying age thresholds.(2) In addition, we used the same cut-off in our previous work on mental healthcare costs in the Netherlands to ensure consistency. (3) In that earlier work, we also conducted an exploratory analysis using smaller age bands.(3) These analyses showed that mental healthcare costs were lower for individuals aged 18–19 compared to those aged 20–34, while costs within the 20-34 age range were relatively stable across the smaller intervals. We hypothesized that the observed differences -particularly between those aged 18-19 and older groups- may reflect structural features of the Dutch mental health system. Specifically, those under the age of 18 are served by different care organizations than those aged 18 and older, which can sometimes lead to discontinuities in care at this transition point.*

*As we previously conducted this analysis for total mental healthcare costs, we did not repeat it in the current paper. In response to the Reviewer’s suggestion, however, we have revised the manuscript to clarify our rationale for the chosen cut-off. We have added the following sentence to the methods section:*

*“In the absence of a universally agreed-upon definition of young adulthood, we chose this cut-off for young adulthood in line with a previous report from the Netherlands on the rise in common mental health disorders to facilitate comparability.” [lines 95-97]*

2. In the Discussion section, the manuscript effectively reports statistically significant differences in mental healthcare expenditures across groups. However, it remains unclear what the practical implications of these differences are from a clinical or policy perspective. Adding interpretation on the practical significance of your findings would enhance the utility of the results for policymakers and clinicians.

*Response: We thank the reviewer for this suggestion. To address this point, we have added the following to the section on gender in the discussion*:

*“Our findings regarding gender could prompt clinicians to evaluate whether the care they offer to young people differs based on gender. The lower expenditures among young men, in particular, could have implications for policies aimed at improving accessibility and engagement with care for this group.” [line 271-274]*

*We have also added the following sentence to the paragraph on the overall rise in costs among youth:*

*“**This finding could serve as an economic incentive for policymakers to invest in prevention strategies and address the underlying causes of mental distress in youth.” [line 277-278]*

*We decided to keep this discussion section concise, as we have explored the clinical and policy implications of these trends more extensively in a previous publication, and we wished to avoid redundancy in the current manuscript. (3)*

3. In the Discussion section, the authors interpret the rising mental healthcare expenditures among younger adults as indicative of worsening mental health in this group. While this is a reasonable and important interpretation, I wonder if the authors have also considered the possibility that the increase reflects a greater willingness to seek help, possibly due to reduced stigma or increased awareness. A brief acknowledgment of this alternative explanation, along with any supporting literature if available, could offer a more nuanced perspective.

*Response: We thank the reviewer for this suggestion. While we had already mentioned this explanation in the section on gender differences, we agree that it is important to explicitly acknowledge it in the context of age-related trends as well. To address this, we have added the following to the discussion section:*

*“It is also possible that the observed increase in mental healthcare costs among younger adults reflects greater help-seeking behaviour. This is supported by studies showing more positive attitudes toward mental health among younger people and a rise in the number of young individuals accessing services over time. (6–8) Additionally, mental distress may have become increasingly medicalized in recent years, which could have contributed to younger generations’ being more open to using mental health services compared to older generations. (9–11)” [lines 291-297]*

**References**

1.	ten Have M, Tuithof M, van Dorsselaer S, Schouten F, Luik AI, de Graaf R. Prevalence and trends of common mental disorders from 2007-2009 to 2019-2022: results from the Netherlands Mental Health Survey and Incidence Studies (NEMESIS), including comparison of prevalence rates before vs. during the COVID-19 pandemic. World Psychiatry. 2023 Jun 1;22(2):275–85.

2.	United Nations. Global issues: Youth [Internet]. 2023 [cited 2024 Jan 31]. Available from: https://www.un.org/en/global-issues/youth

3.	Dijkstra L, Gülöksüz S, Batalla A, van Os J. Young adults, particularly young women, account for an increasingly large share of Dutch mental healthcare expenditure over the period between 2015 and 2021. Epidemiol Psychiatr Sci. 2024 Oct 11;33:e48.

4.	Zorginstituut Nederland. Zorgcijfersdatabank [Healthcare figures databank] [Internet]. 2025 [cited 2025 Jun 18]. Available from: https://www.zorgcijfersdatabank.nl/

5.	Nederlandse Zorgautoriteit. Informatiekaart Zorgprestatiemodel per 2022 [Informationcard “Zorgprestatiemodel” from 2022] [Internet]. 2021 [cited 2024 Jan 31]. Available from: https://puc.overheid.nl/nza/doc/PUC_646523_22/#:~:text=Per%202022%20gaan%20we%20in,en%20controleerbaar%20voor%20de%20pati%C3%ABnt.

6.	Clarkin J, Heywood C, Robinson LJ. Are younger people more accurate at identifying mental health disorders, recommending help appropriately, and do they show lower mental health stigma than older people?: Age differences in mental health disorder recognition. Ment Health Prev. 2024 Dec 1;36.

7.	Lipson SK, Lattie EG, Eisenberg D. Increased rates of mental health service utilization by U.S. College students: 10-year population-level trends (2007-2017). Psychiatric Services. 2019 Jan 1;70(1):60–3.

8.	Oswalt SB, Lederer AM, Chestnut-Steich K, Day C, Halbritter A, Ortiz D. Trends in college students’ mental health diagnoses and utilization of services, 2009–2015. Journal of American College Health. 2020 Jan 2;68(1):41–51.

9.	Aneshensel CS, Phelan JC, Bierman A. Handbook of the Sociology of Mental Health [Internet]. 2nd ed. 2013. Available from: http://www.springer.com/series/6055

10.	Beeker T, Mills C, Bhugra D, te Meerman S, Thoma S, Heinze M, et al. Psychiatrization of Society: A Conceptual Framework and Call for Transdisciplinary Research. Front Psychiatry. 2021 Jun 4;12.

11.	Foulkes L, Andrews JL. Are mental health awareness efforts contributing to the rise in reported mental health problems? A call to test the prevalence inflation hypothesis. New Ideas Psychol. 2023 Apr 1;69.
